# Ag–Co ferrite-based magnetic polymeric composite film: a breakthrough in cationic dye remediation for sustainable environment†

**DOI:** 10.1039/d4ra06315e

**Published:** 2024-11-15

**Authors:** Nafisa Tabassum, Raamisa Anjum, Papia Haque, Md. Sahadat Hossain, Mashrafi Bin Mobarak, Md. Saiful Quddus, Fariha Chowdhury, Lutfor Rahman, Dipa Islam, Samina Ahmed, Monika Mahmud

**Affiliations:** a Department of Applied Chemistry and Chemical Engineering, University of Dhaka Dhaka-1000 Bangladesh papiahq@du.ac.bd; b Institute of Glass and Ceramic Research and Testing (IGCRT), Bangladesh Council of Scientific and Industrial Research (BCSIR) Dr. Qudrat-i-Khuda Road, Dhanmondi Dhaka-1205 Bangladesh shanta_samina@yahoo.com monika0197@gmail.com; c BTRI, Bangladesh Council of Scientific and Industrial Research (BCSIR) Dr. Qudrat-i-Khuda Road, Dhanmondi Dhaka-1205 Bangladesh; d BCSIR Laboratories Dhaka, Bangladesh Council of Scientific and Industrial Research (BCSIR) Dr. Qudrat-i-Khuda Road, Dhanmondi Dhaka-1205 Bangladesh

## Abstract

The deployment of magnetically responsive and polymeric materials to remove dyes that are hazardous in aquatic environments has profoundly revolutionized environmental sustainability. This study focuses on removing the hazardous cationic Malachite Green (MG) dye from solutions, employing a novel magnetic composite film as an adsorbent, designated as Ag_0.2_Co_0.8_ Fe_2_O_4_ (ACFCeP). The composite was synthesized *via* solvent casting, incorporating Ag_0.2_Co_0.8_ Fe_2_O_4_ nanoparticles and CeO_2_ into a cellulose acetate/polyvinylpyrrolidone (CA/PVP) polymer matrix. The Ag_0.2_Co_0.8_Fe_2_O_4_ nanoparticles were synthesized by a co-precipitation method. Comprehensive characterization of the synthesized composite was conducted using techniques, such as Fourier transform infrared spectroscopy (FT-IR), X-ray diffraction (XRD), X-ray photoelectron spectroscopy (XPS), field emission scanning electron microscopy (FE-SEM), and vibrating sample magnetometer (VSM). The Ag-doped cobalt ferrite component retained a strong hysteresis loop within the final composite, even when blended with the CA/PVP polymer, preserving the robust magnetic properties that facilitate the easy removal of the composite post-treatment without secondary pollution. Additionally, the mesoporous structure of the composite effectively aids in the adsorption mechanism. The isothermal study shows that both linear Langmuir isotherm and Freundlich isotherm are well fitted with *R*^2^ values of 0.99 and 0.97, respectively. The linear Langmuir maximum adsorption capacity, *q*_max_, is 45.66 mg g^−1^ at pH 7. The kinetic studies of the composite resemble the pseudo-second-order kinetic model, reaching adsorption equilibrium within 70 min for a 100 ppm MG dye concentration. The composite film exhibits excellent reusability, maintaining high removal efficiency over three cycles. Overall, the ACFCeP composite film showcases excellent dye removal capabilities, a fast adsorption rate, and satisfactory magnetic properties and offers a sustainable solution for environmental pollution, thus contributing to ecosystem preservation through efficient recycling and reuse in dye adsorption applications.

## Abstract 2

The deployment of magnetically responsive and polymeric materials to remove dyes that are hazardous in aquatic environments has profoundly revolutionized environmental sustainability.

## Graphical abstract

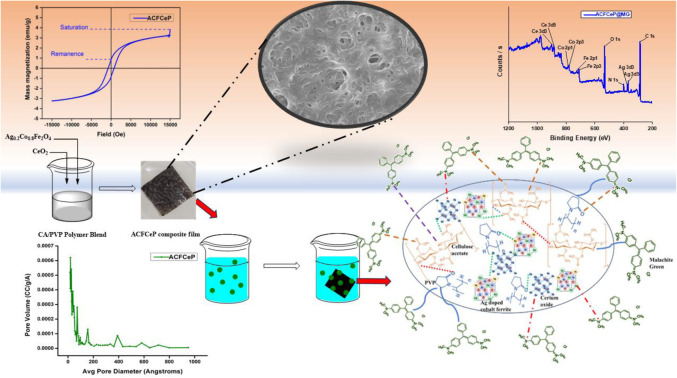

## 1 Introduction

In today's industrial landscape, dyes are ubiquitously harnessed across a diverse array of sectors—such as textiles, paper manufacturing, tanneries, plastics, paints, and agriculture—chiefly for their vibrant pigmentation attributes.^1^ This led to the development of 10 000 different synthetic dyes and global annual synthetic dye production of 7 × 10^5^ tons, out of which the textile industries discharge 3600 tons of concentrated dye waste in the natural water resources.^2^ The persistent, non-biodegradable synthetic dyes are responsible for making the water unsuitable for drinking, while the demand for freshwater is expected to increase from the current 4500 billion cubic meters per year to 6900 billion cubic meters by 2030.^2,3^ Moreover, the presence of dyes significantly alters various aquatic environmental parameters like BOD and COD, causing damage to the aquatic food chain, photosynthesis and exerting detrimental effects on aquatic life.^4–6^ Additionally, dyes can cause a multitude of health issues, affecting the nervous system, skin, liver, kidneys, reproductive system, and enzymatic functions within the human body.^6^

Among synthetic dyes, Malachite green (MG) is extensively employed in aquaculture and other industries. Initially, it was effective as a parasiticide, fungicide, and antiprotozoan agent. However, subsequent research revealed that MG is highly toxic, with its toxicity escalating with increased temperature, time, and concentration.^7^ Consequently, treating wastewater containing MG dye is imperative to safeguard both aquatic and terrestrial life from its acute toxicity.

Several biological, physical and chemical methods exist for the treatment of wastewater, among which adsorption stands out as a physical process. It is highly effective, straightforward, cost-efficient, energy-conserving, and easy to operate.^8^ Ongoing research aims to develop superior, eco-friendly, and more efficient adsorbents for maximum dye removal. In pursuit of this goal, numerous composite adsorbents are being developed to combine the most beneficial properties into a single material.

Recently, magnetic nanoparticles, particularly spinel ferrite nanoparticles (MFe_2_O_4_; M can be Fe, Ni, Co, Mn, Zn, *etc.*), have gathered remarkable attention because of their high surface area, exceptional magnetic properties and active sites, resulting in enhanced adsorption capacity and broad applicability.^9,10^ Spinel ferrite (SF) has the chemical formula AB_2_O_4_, where A occupies the tetrahedral site (*e.g.*, Pb, Mg, Cr, Mn) and B occupies the octahedral site (*e.g.*, Fe, Al, Cu).^11^ The cations at both sites are connected to oxygen atoms tetrahedrally and octahedrally, respectively.^9^

Among the three types of spinel ferrites—normal, inverse, and mixed—CoFe_2_O_4_ is an inverse spinel ferrite. It is a significant SF as it is chemically stable and has intrinsic magnetic, electrical, and mechanical properties.^12^ Cobalt ferrite possesses a mesoporous structure with moderate saturation magnetization, making it suitable as both an adsorbent and a photocatalyst for pollutant removal.^13–15^ Studies have demonstrated that hydrothermally synthesized CoFe_2_O_4_ exhibits one of the highest adsorption capacities to remove Congo red dye from solutions.^16,17^ Post-adsorption, removing adsorbents from the solution is crucial to prevent further contamination. Conventional separation methods like filtration, sedimentation, or coagulation are laborious and expensive.^18^ In contrast, magnetic separation is simpler, more economical, and effective, as it uses an external magnetic field to remove the adsorbent from the effluent, ensuring no additional pollution in the treated water.

Recent advancements have seen techniques such as surface modification, metal ion doping and nanocomposite preparation to enhance the adsorption capacity of ferrite nanoparticles.^19^ Doping cobalt ferrite with various metals and non-metals has been shown to improve coercive force and specific surface area.^20,21^ Significant interest is placed on Ag nanoparticles due to their high conductive nature, good catalytic performance and wide range of antibacterial actions.^22^ Many works have proved the enhancement of the adsorption capacity for both cationic and anionic dyes after the addition of Ag nanoparticles to materials, including CoFe_2_O_4_.^23–25^

Moreover, as a rare earth metal oxide, cerium oxide is characterized by its cubic crystal structure and has demonstrated a remarkable ability to enhance dye adsorption capacity. This enhancement is attributed to its high surface area, affordability, water solubility, catalytic properties, and chemical reactivity.^26–28^ Thus, integrating cerium oxide into composite films can markedly boost their dye adsorption efficiency.

Other than the nanoparticles, natural polymers are gaining attention due to their biodegradability, non-toxicity, flexibility, and eco-friendliness.^29^ Cellulose acetate (CA) is one of these and has been found to have wide applicability as an adsorptive polymer matrix because of its biocompatibility, low cost, film-forming ability and simple preparation.^30–32^ It has been functionalized with different organic and inorganic materials and showed better performance in removing different dyes.^33,34^ A study has also reported the fabrication of ferrite in CA to adsorb methylene blue.^35^ The film formation ability of CA facilitates better adsorption by improving the surface area.^32^

Even synthetic polyvinylpyrrolidone (PVP), a non-ionic, biodegradable, non-toxic, hydrophilic polymer, is also known for its excellent film-forming properties.^36–38^ PVP binds with nanoparticles, preventing aggregation and providing stability and effectiveness.^39–41^ It also increases the dye adsorption capacity by creating a porous film.^42^

This research endeavors to engineer an innovative composite film that amalgamates Ag-doped cobalt ferrite, cerium oxide, cellulose acetate (CA), and polyvinylpyrrolidone (PVP). By proposing a heterostructured composite film, we aim to prevent the aggregation of powdered materials, thereby enhancing its efficacy. Comprehensive analyses, including XPS to observe the interactions and crosslinking, along with N_2_ gas adsorption–desorption for assessing the surface area and pore size, have been meticulously conducted. Furthermore, VSM was utilized to determine the magnetic responsiveness of the composite. Additionally, adsorption isotherms and kinetic models have been scrutinized to elucidate the adsorption method. Emphasizing the magnetic properties of cobalt ferrite, this study highlights its pivotal role in environmental pollution mitigation. The reusability of the composite across multiple cycles is also rigorously assessed, underscoring its potential for sustainable and eco-friendly applications in protecting our ecosystem.

## 2 Materials & methods

### 2.1 Materials

Ferric nitrate [Fe(NO_3_)_3_·9H_2_O], cobalt nitrate [Co(NO_3_)_2_·6H_2_O], silver nitrate (AgNO_3_) and sodium hydroxide (NaOH) were obtained from Merck, Germany. Hydrochloric acid (HCl), cellulose acetate, polyvinylpyrrolidone (PVP), cerium oxide (CeO_2_) and Malachite Green were purchased from Sigma-Aldrich. Acetone and *N*,*N*-dimethylformamide (DMF) were also purchased from Sigma-Aldrich to prepare the cellulose acetate and PVP solutions.

### 2.2 Methods

#### 2.2.1 Synthesis of cobalt ferrite (CF) and Ag-doped cobalt ferrite (ACF) nanoparticles

Cobalt ferrite (CoFe_2_O_4_) nanoparticles were synthesized by following the traditional co-precipitation method.^11^ In this method, 0.4 M 50 mL Fe(NO_3_)_3_·9H_2_O and 0.2 M 50 mL Co(NO_3_)_2_·6H_2_O salt solution were prepared and mixed. Dropwise addition of 3 M NaOH solution in it accelerated the nucleation process. This process continued under constant stirring until the pH became 12. The reaction temperature required an amount of deionized water to make the particles free from unwanted sodium and nitrate components. The CF nanoparticles were then dried at 100 °C for around 10 h, and the dried products were annealed at 600 °C for 5 h. The reaction scheme is given in eqn (1).

1 Co(NO_3_)_2_·6H_2_O + 2Fe(NO_3_)_3_·9H_2_O + NaOH → CoFe_2_O_4_ + 8NaOH + 8NaNO_3_ + 28H_2_O

**Scheme 1:**
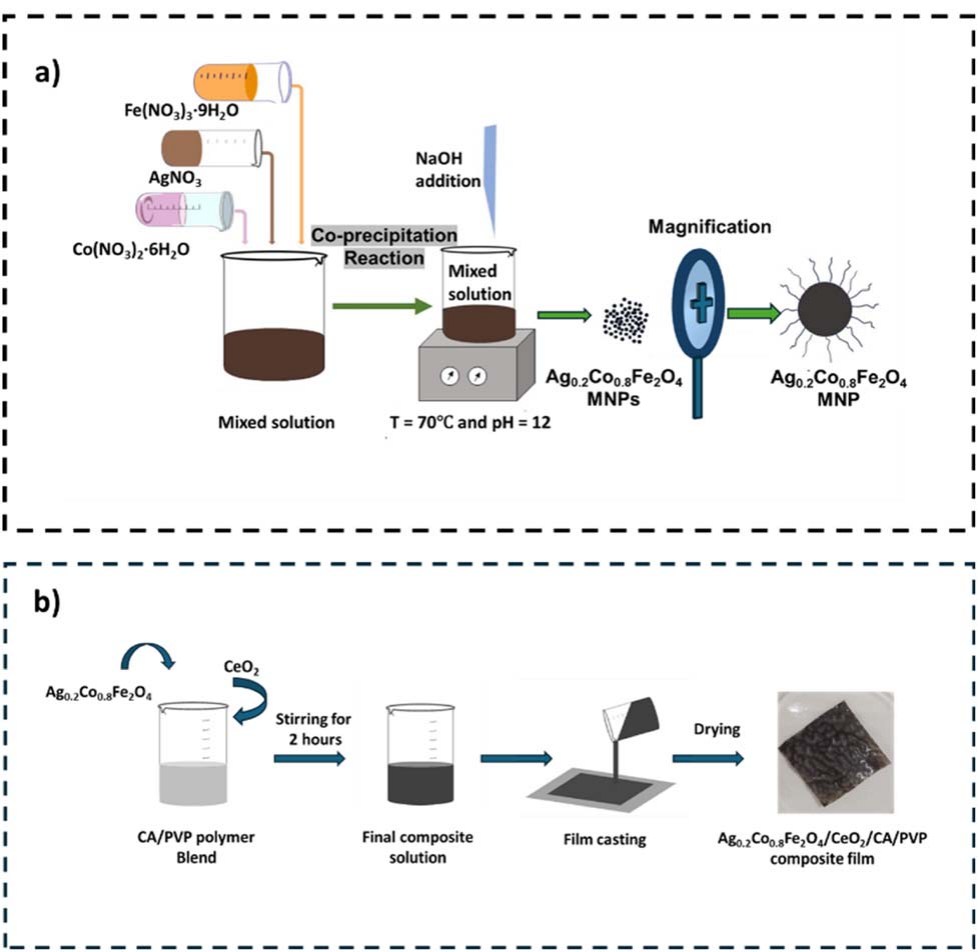
Preparation of the Ag-doped cobalt ferrite (a); preparation of the Ag_0.2_Co_0.8_Fe_2_O_4_/CeO_2_/CA/PVP (ACFCeP) composite film (b).

Ag_0.2_Co_0.8_Fe_2_O_4_ nanoparticles (Ag_*x*_Co_1−*x*_Fe_2_O_4_; *x* = 0.2) were synthesized in the same manner described previously by adding 0.4 M 50 mL Fe(NO_3_)_3_·9H_2_O and 0.16 M 50 mL Co(NO_3_)_2_·6H_2_O solution with 0.04 M 25 mL AgNO_3_ salt solutions. The preparation procedure is represented in Scheme 1a.

#### 2.2.2 Preparation of the Ag_0.2_Co_0.8_Fe_2_O_4_/CeO_2_/CA/PVP composite film

A 2% solution of cellulose acetate (CA) was prepared by dissolving it in 10 mL of acetone/DMF (4 : 1, v/v) under continuous stirring for 30 min in a fume hood. Similarly, 2% polyvinylpyrrolidone (PVP) was dissolved in 10 mL of acetone/DMF (4 : 1, v/v) and also stirred for 30 min in the fume hood. These two solutions were then mixed together and stirred continuously for 1 hour to ensure homogeneity. The stirring was conducted inside a fume hood to ensure the safe exhaustion of the evaporated solvent. Next, 12.5 wt% of Ag_0.2_Co_0.8_Fe_2_O_4_ (ACF) nanoparticles and 2 wt% of CeO_2_ powder (based on the total weight of solid polymers) were added to the CA/PVP mixture. A further stirring for 2 h was continued by placing the solution in a fume hood at room temperature. The resulting polymer solution, now containing finely dispersed ACF nanoparticles and cerium oxide, was carefully poured into a 60 mm diameter Petri dish. It was then naturally air dried at room temperature at 22 ± 5 °C and 35 ± 5% relative humidity in a well-ventilated area for 24 h. The remaining organic solvent slowly evaporated during this period, generating pores in the film. After 24 h, the dried Ag_0.2_Co_0.8_Fe_2_O_4_/CeO_2_/CA/PVP (ACFCeP) composite film was obtained by carefully peeling it off the Petri dish. The resulting film had a uniform thickness. As a comparison, a blank CA/PVP film (without ACF and CeO_2_) was also prepared to evaluate the dye removal efficiency of the composite. Scheme 1b illustrates the preparation process of the composite film.

#### 2.2.3 Preparation of the standard Malachite Green dye (MG dye) solution

500 ppm Malachite Green stock solution was prepared by adding 250 mg of MG dye into 500 mL deionized water in a 500 mL volumetric flask. The molecular weight of MG is 364.911 g mol^−1^. The dilution of the stock solution was carried out in exact proportions to prepare the experimental dye solutions of desired initial concentrations. The concentration of Malachite Green in the aqueous solutions was obtained at a *λ*_max_ of 617 nm using a UV-1900i (Shimadzu Corporation, Japan) spectrophotometer. The pH was adjusted using 0.1 M NaOH and 0.1 M HCl solutions.

#### 2.2.4 Batch adsorption study for the removal of dye

To evaluate the removal efficiency of Malachite Green (MG) dye by the nanocomposite film, batch adsorption experiments were conducted. To study the effect of solution pH on MG adsorption, 50 mg of the composite was introduced into a 50 ppm dye solution across a pH range of 2 to 8, with a contact time of 90 min and a shaking speed of 200 rpm. The pH was maintained using 0.1 M NaOH or 0.1 M HCl. To investigate the effect of adsorbent dosage, various doses of the ACFCeP adsorbent ranging from 10 to 100 mg were added to 30 mL of 50 ppm MG dye solution, with a shaking speed of 200 rpm for 90 min. To study the influence of the initial dye concentration, solutions with MG dye concentrations varying from 20 to 100 ppm were prepared. 50 mg of the adsorbent was introduced into 30 mL of the dye solution at pH 7, maintaining a contact time of 90 min and a shaking speed of 200 rpm. To examine the effect of contact time, MG dye solutions of 20, 60, and 100 ppm were prepared, and 50 mg of the adsorbent was added to 30 mL of the solution. The dye solutions were placed in a reciprocating shaker operating at 200 rpm, with the pH held constant at 7 and a contact time of 90 min.

At equilibrium, the solutions were allowed to settle, and the MG-loaded composites were separated. The remaining equilibrium concentration of MG in the solutions was calculated by measuring the absorbance at 617 nm (*λ*_max_ of MG) in a UV-vis spectrophotometer and *via* the calibration curve of MG.

The removal percentage of the dye was determined using the following formula:2
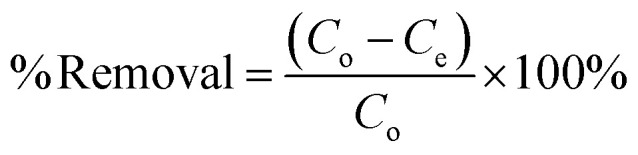
Here, *C*_o_ and *C*_e_ represent the initial and equilibrium concentrations of MG (mg L^−1^), respectively.

The amount of adsorbed Malachite Green per gram of the adsorbent (*q*_e_) was calculated according to the following equation:3
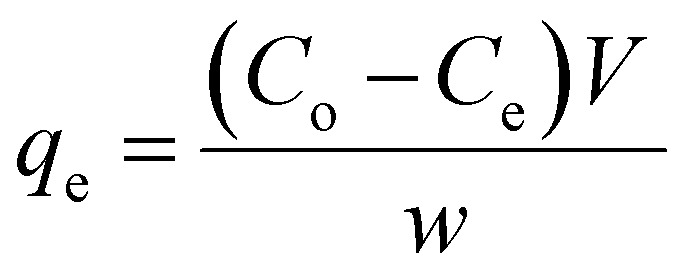
Here, *q*_e_ indicates the adsorption capacity (mg g^−1^), *V* denotes the volume of the dye solution (L), *w* represents the amount of adsorbent (mg), *C*_o_ and *C*_e_ denote the initial and equilibrium concentrations of MG (mg L^−1^), respectively.

#### 2.2.5 Reusability study

Reusability analysis of the composite film was performed by regenerating the film after dye adsorption with 0.1 M HCl, which removes the adsorbed MG dye from the film as MG is a basic cationic dye. Three sequential cycles of adsorption and desorption were performed to study the reusability of the film. The dye-adsorbed film was treated with HCl for 30 min at 220 rpm and washed with deionized water to obtain a neutral pH. The reusability study for multiple-cycle dye adsorption was performed by adding the regenerated composite film in a freshly made dye solution of optimized conditions. The used composite film was removed from the solutions after adsorption, and the residual concentration of the dye solution was determined using a UV-vis spectrometer. The used-up composite film was again regenerated using the exact same technique. Thus, the reusability of the composite film was analyzed according to three sequential adsorption–desorption cycles.

### 2.3 Material characterization

#### 2.3.1 Fourier-transform infrared (FT-IR) spectroscopy

Fourier transform infrared (FT-IR) spectra were used to determine the functional groups present in the samples. The spectra were measured using an IR Prestige-21 spectrophotometer (Shimadzu Corporation, Kyoto, Japan), maintaining the wavenumber range from 4000 to 400 cm^−1^, and the resolution of the spectra was 4 cm^−1^.

#### 2.3.2 X-ray diffractometer (XRD) analysis

XRD analysis was performed in order to understand the crystalline phase of samples. An X-ray diffractometer (Ultima IV, Rigaku Corporation, Japan) was used in this regard at room temperature. A Cu tube was operated at 40 kV and 40 mA to apply the Cu *K*_α_ radiation (*λ* = 0.154 nm). The measurement of the XRD patterns was done in the continuous scanning mode, maintaining a scan speed of 3° min^−1^ and in the 5–90° scan range. The basal spacing of the crystalline samples was calculated using Bragg's law.

#### 2.3.3 X-ray photoelectron spectrometer (XPS) analysis

The chemical composition of the composite film was studied using an X-ray photoelectron spectrometer (K-Alpha). An Al K-Alpha monochromatic X-ray source of 1486.69 eV was used, operating at 15 kV and 10 mA for the initial survey and core-level spectra of the sample. The residual pressure of the analysis chamber was kept below 10^−8^ torr.

#### 2.3.4 Field emission scanning electron microscopy (FE-SEM) analysis

The shapes and surface morphologies of CF, ACF, CA/PVP and ACFCeP were studied using a field emission scanning electron microscope, FESEM (JSM-7610F), operating at 5 kV voltage. The JSM-7610F is a semi-in-lens field emission scanning electron microscope with ultra-high resolution. Tests were conducted after the samples were sputter-coated with platinum.

#### 2.3.5 N_2_ gas adsorption isotherms using the BET model

The adsorption–desorption isotherms using nitrogen gas were performed at 196 °C using BET-201-A to determine the surface texture properties of the ACFCeP composite film. The specific surface area was analyzed using the Brunauer–Emmett–Teller (BET) model, and the *P*/*P*_0_ data range was set between 0.05 and 0.35. The Barrett–Joyner–Halenda (BJH) model was further used to measure the pore volume and pore diameter of the sample.

#### 2.3.6 Vibrating sample magnetometer (VSM) analysis

A vibrating sample magnetometer [Physical Property Measurement System (PPMS), DynaCool, Quantum Design] was used to study the magnetic properties of the prepared samples. A magnetic field of −20 000 to 20 000 Oe was applied at room temperature for the analysis.

#### 2.3.7 Isotherm and kinetics study

Langmuir, Freundlich, Dubinin–Radushkevich (D–R) and Temkin isotherms are used in this study to understand the distribution of the adsorbate MG dye molecule on the ACFCeP adsorbent surface.

The linear and non-linear forms of Langmuir isotherms are represented by eqn (4) and (5), respectively.^43^4
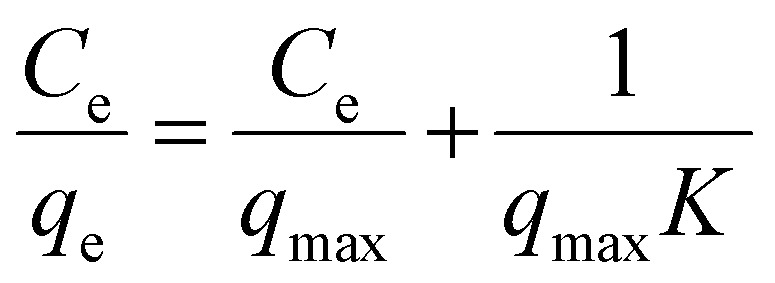
5
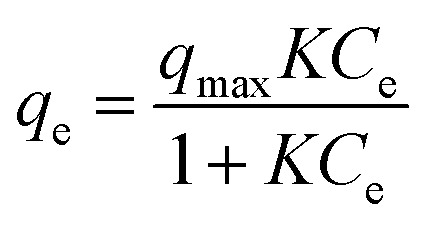
Here, *C*_e_ (mg L^−1^) denotes the adsorbate equilibrium concentration, *q*_e_ (mg g^−1^) represents the quantity of adsorbate adsorbed per gram of the adsorbent at equilibrium, *q*_max_ (mg g^−1^) indicates the maximum adsorption capacity and *K* (L mg^−1^) is called the Langmuir isotherm constant, which represents the binding strength between adsorbents and adsorbates.

Freundlich isotherms in the linear and non-linear forms are presented by eqn (6) and (7), respectively.^44^6
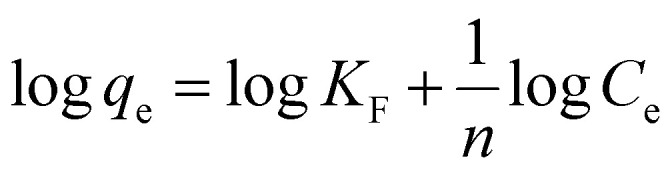
7
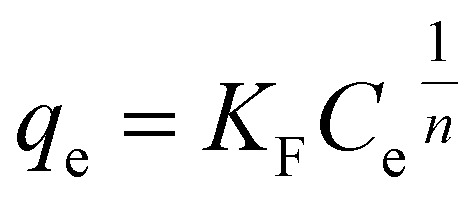
Here, *K*_F_ (mg g^−1^) is known as the Freundlich isotherm constant, which is related to the relative adsorption capacity of the adsorbent, *n* represents the adsorption intensity, *C*_e_ (mg L^−1^) denotes the adsorbate equilibrium concentration and *q*_e_ (mg g^−1^) denotes the amount of dye adsorbed per gram of the adsorbent at equilibrium.

Dubinin–Radushkevich isotherm is used to determine whether the adsorption process undergoes physisorption or chemisorption. The linear and non-linear forms of the D–R isotherm are shown in eqn (8) and (9), respectively.^45^

8 ln Q_e_ = ln Q_m_ − *β*_DR_*ε*^2^

9 *Q*_e_ = *Q*_m_e^*β*_DR_*ε*^2^^

10
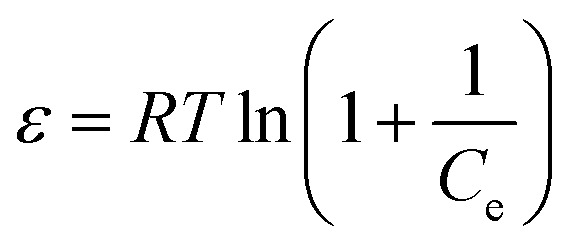
11
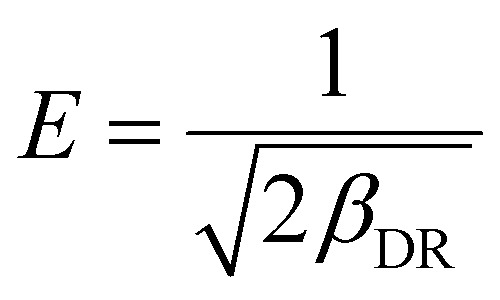
In the above equations, *Q*_m_(mg g^−1^) denotes the D–R maximum adsorption capacity, and *β*_DR_ (mol^2^ kJ^−2^) represents the mean adsorption energy. *ε* indicates the Polanyi potential, which can be determined by eqn (10) where *R* is the gas constant (8.31 J mol^−1^ K^−1^), *T* represents the absolute temperature and *C*_e_ indicates the equilibrium adsorbate concentration. *E* is the free energy of adsorption per molecule, which can be determined by eqn (11).

Eqn (12) and (13) represent the linear and non-linear forms of Temkin equations, respectively.^46^

12 *q*_e_ = *A* ln *K*_T_ + *A* ln *C*_e_

13 *q*_e_ = *A* ln(*K*_T_*C*_e_)

14
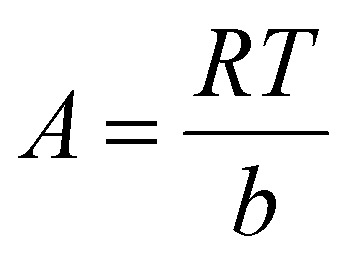
In the above equations, *A* represents the unitless Temkin isotherm constant; *b* (J mol^−1^) indicates the heat of adsorption; *K*_T_ (L g^−1^) denotes the Temkin equilibrium binding constant. *R* and *T* in eqn (14) indicate the gas constant (8.31 J mol^−1^ K^−1^) and absolute temperature, respectively.

The dye adsorption rate is dependent on both the contact time and diffusion process between the adsorbate and the adsorbent.^47^ The adsorption process happens in two specific steps. In the first step, molecules migrate from the aqueous dye solution towards the adsorbent, and in the next step, diffusion on the adsorbent surface occurs.^48^ To understand the adsorption kinetics of the ACFCeP film, Lagergren pseudo-first-order equation, pseudo-second-order equation and Elovich model were assessed. The linear and non-linear pseudo-first-order equations are given in eqn (15) and (16), respectively.^49^

15 ln(*q*_e_ − *q*_t_) = ln *q*_e_ + *K*_1_*t*

16 *q*_t_ = *q*_e_(1 − e^−*K*_1_*t*^)

Here, *q*_e_ and *q*_t_ indicate the adsorbent's adsorption capacity (mg g^−1^) at equilibrium and at any time *t*, respectively, and *K*_1_ (min^−1^) presents the rate constant of the pseudo-first-order adsorption process.

The linear and non-linear forms of the pseudo-second-order model are shown in eqn (17) and (18), respectively.^50^17
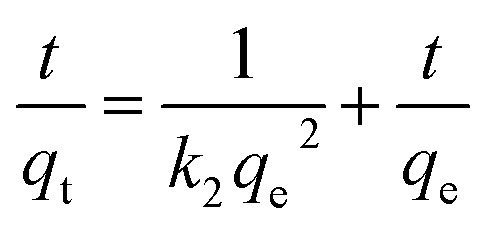
18
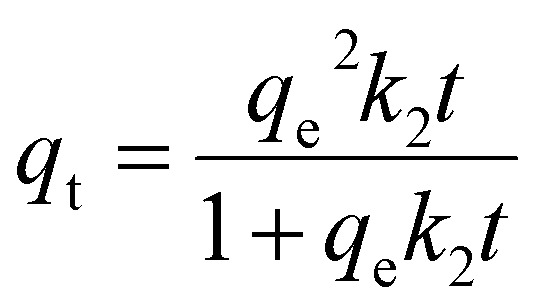
Here, *k*_2_ (g mg^−1^ min^−1^) denotes the rate constant of the pseudo-second-order adsorption, *q*_e_ (mg g^−1^) and *q*_t_ (mg g^−1^) describe the quantity of the dye adsorbed at equilibrium and at time *t*, respectively.

Eqn (19) and (20) present the linear and non-linear Elovich kinetic models, respectively.^51^19
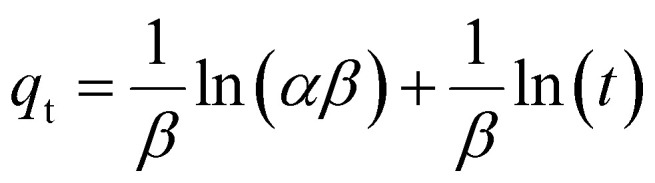
20
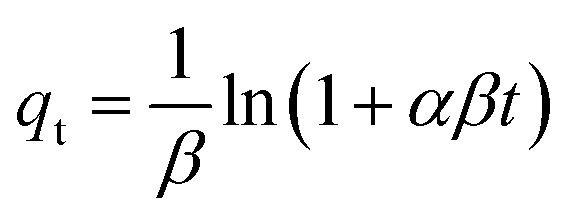
In the above equations, *q*_t_ (mg g^−1^) denotes the adsorption capacity, *α* (mg g^−1^ min^−1^) indicates the rate of chemisorption in the case of zero coverage and *β* (mg g^−1^) represents the maximum coverage of the adsorbate surface.

## 3 Results and discussion

### 3.1 XRD and FTIR analysis

**Fig. 1:**
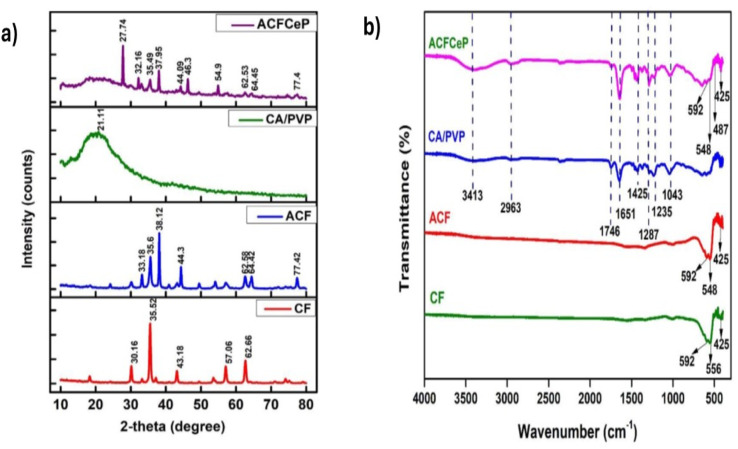
XRD pattern (a) and FTIR spectra (b) of cobalt ferrite, Ag-doped cobalt ferrite, CA/PVP blend and ACFCeP composite.

The XRD pattern of the synthesized cobalt ferrite (CF), Ag-doped cobalt ferrite (ACF), CA/PVP and Ag_0.2_Co_0.8_Fe_2_O_4_/CeO_2_/CA/PVP samples are displayed in Fig. 1a. The peaks were sharp and intense, indicating the crystalline structure and high purity of spinel CoFe_2_O_4_ (CF). The XRD pattern of cobalt ferrite showed its characteristic peak at 2*θ* = 35.52° of the (311) planes and resembled the crystalline FCC structure (JCPDS card no. 22-1086).^11,52^ The XRD pattern of Ag_0.2_Co_0.8_Fe_2_O_4_ (ACF) exhibits strong sharp peaks, presenting the crystallinity of ACF. The characteristic peak of Ag is also found at 2*θ* = 38.12°, corresponding to the (111) plane, confirming the presence of Ag.^53^ From the CA/PVP XRD pattern, a broad diffraction spectrum at 2*θ* = 18–22° is visible. This is due to the amorphous structure of the polymer blend and the replacement of hydroxide groups of cellulose by acetyl groups.^54^ From the diffraction pattern of the composite material ACFCeP, all the dominant peaks of Ag-doped cobalt ferrite are present. Besides that, the characteristic peak of CeO_2_ is observed at 2*θ* = 27.74° in the ACFCeP XRD pattern.^55^ The highly intense peaks indicate the crystallinity of the composite film after incorporating ACF and CeO_2_ in the amorphous CA/PVP polymer blend. The low intensity of the characteristic peak of cobalt ferrite at 2*θ* = 35.52° in the XRD of the composite material is because of the polymer matrix coating on cobalt ferrite particles, affecting the crystallinity of cobalt ferrite.^56^ The characteristic peak of Ag at 2*θ* = 38.12° has been shifted to 2*θ* = 37.95°. This change is due to expansion of the interplanar distance caused by the doping effect of CoFe_2_O_4_ nanoparticles and CeO_2_ as well as the capping effect of PVP present in the polymeric matrix. The interaction of the substituted CoFe_2_O_4_ nanoparticles and CeO_2_ with the Ag nanoparticles caused strain in the crystal structure.^57,58^ PVP, on the other hand, as a capping agent, can form coordinate bonds with silver through its carbonyl group, which affects the atomic arrangement on the nanoparticle surface and changes the lattice structure of Ag.^59^ These two effects combined altered the crystal structure of silver and are responsible for the small shift in peaks.^60^

The FTIR spectra of cobalt ferrite, Ag-doped cobalt ferrite, CA/PVP and the ACFCeP composite are demonstrated in Fig. 1b. In the spectrum of cobalt ferrite, the higher frequency absorption peak at 592 cm^−1^ and 556 cm^−1^ indicates the vibration of the tetrahedral metal oxide (M−O, M = Fe, Co) site and the lower frequency absorption peak at 425 cm^−1^ indicates the vibration of octahedral M−O of the spinel structure of cobalt ferrite.^61,62^ As Ag was doped in the cobalt ferrite, the characteristic peak of Ag–O is observed at 548 cm^−1^.^63^

In the CA/PVP blend, the stretching vibration of O–H at 3413 cm^−1^, bending of the C–H group at 1425 cm^−1^ and asymmetric stretching vibration of the C–H group at 2963 cm^−1^ resemble cellulose acetate.^64,65^ Moreover, the symmetric stretching of C=O from the COO^−^ bond of CA is observed at 1746 cm^−1^.^54^ The CA/PVP spectrum also shows C–O stretching from the acetate group and stretching of the C–O–C group from cellulose at 1235 cm^−1^ and 1043 cm^−1^, respectively. The bands observed at 1287 cm^−1^ and 1651 cm^−1^ are attributed to the C–N stretching of the pyrrole structure and C=O stretching of the lactam ring from PVP, respectively.^66^

In the spectrum of the composite film, all the characteristic peaks of its components are present, indicating the successful preparation of the composite. Notably, in the ACFCeP composite, the peak observed at 487 cm^−1^ represents the Ce–O group from cerium oxide incorporated into the composite.^67,68^

### 3.2 XPS study

**Fig. 2:**
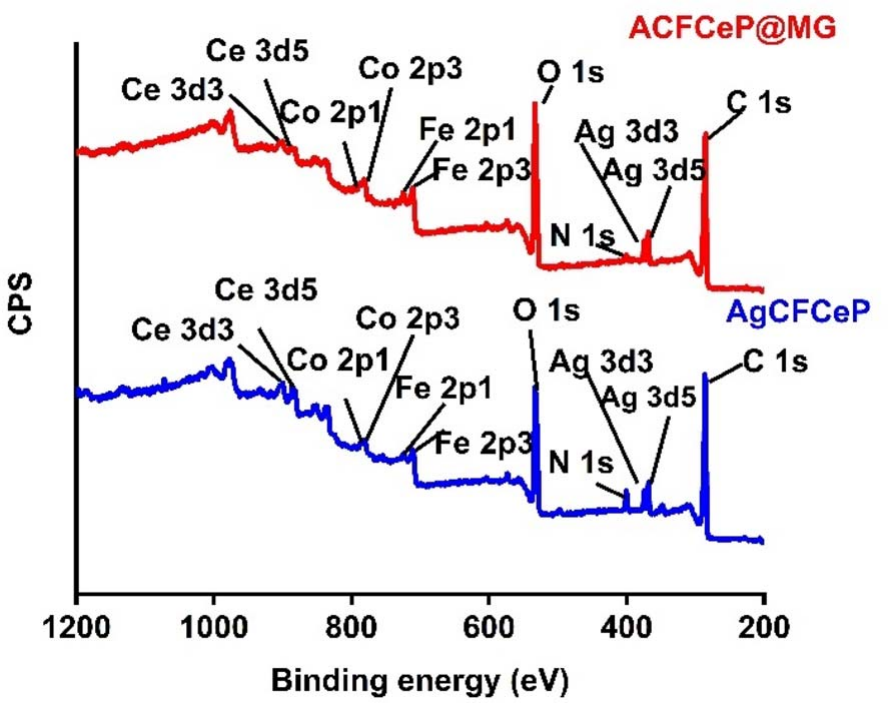
XPS survey spectra of the ACFCeP composite before and after Malachite Green dye adsorption.

The XPS survey spectra of the as-prepared ACFCeP composite before and after MG adsorption are shown in Fig. 2. The presence of C, O, N, Ag, Fe, Co, and Ce on the survey spectra suggests that the composite was formed successfully (Table S1†).

**Fig. 3:**
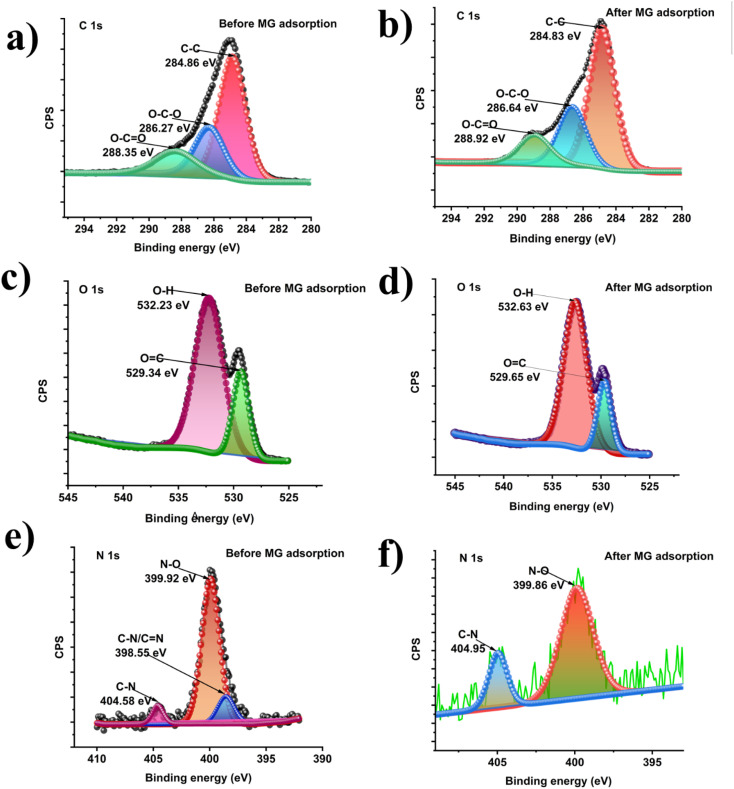
XPS deconvoluted C 1s spectra of the ACFCeP composite before adsorption (a) and after adsorption (b); O 1s spectra of the ACFCeP composite before adsorption (c) and after adsorption (d); N 1s spectra of the ACFCeP composite before adsorption (e) and after adsorption (f).

The major peaks observed around 285.92 eV, 532.87 eV, and 399.9 eV are attributed to the C 1s, O 1s and N 1s spectra, respectively.^69^ The C 1s of the composite were deconvoluted into three peaks placed at 284.86, 286.27, and 288.35, eV (Fig. 3a) allocated to C–C, O–C–O, and O–C=O, respectively.^70,71^ After adsorption, the binding energy shifted to 284.92, 286.64, and 288.82 eV (Fig. 3b) due to the π–π interaction of the dye with the aromatic ring of the composite polymers. The O 1s spectra of the composite show the functional O–H and O=C groups at 532.23 eV and 529.34 eV (Fig. 3c).^71,72^ After dye adsorption, the O 1s is shifted to 532.63 and 529.65 eV (Fig. 3d) due to the electrostatic interaction of the dye with the composite. In Fig. 3e, N 1s spectra before MG adsorption are deconvoluted into three peaks at 399.92, 398.55, and 404.58 for N–O, C–N/C=N, C–N bonds, respectively, whereas after MG adsorption (Fig. 3f), peaks appear at 399.86 and 404.95 and the peak at 398.55 disappeared (Table S2†).

**Fig. 4:**
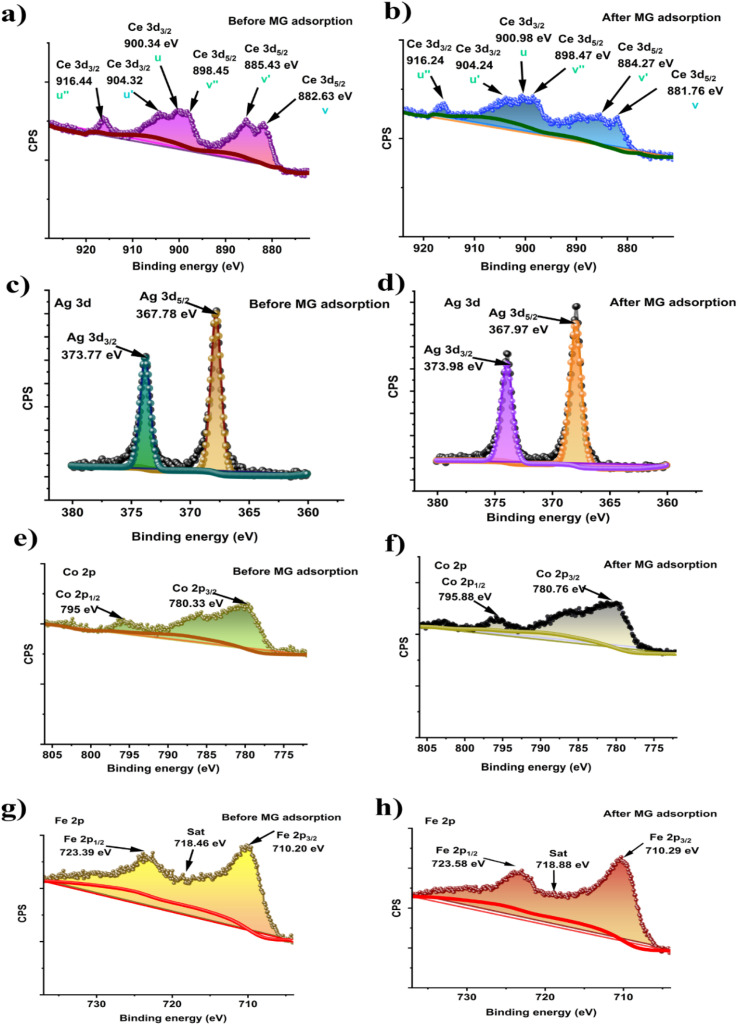
XPS deconvoluted Ce 3d spectra of the ACFCeP composite before adsorption (a) and after adsorption (b); Ag 3d spectra of the ACFCeP composite before adsorption (c) and after adsorption (d); Co 2p spectra of the ACFCeP composite before adsorption (e) and after adsorption (f); Fe 2P spectra of the ACFCeP composite before adsorption (g) and after adsorption (h).

In dye adsorption, nitrogen atoms can donate electrons to the metal centres; therefore, their electron cloud distribution changes, which explains the disappearance of the peak at 398.55 eV. Similar peak disappearance after adsorption is also reported by P. Du *et al.*^73^ Also, the disappearance of the 398.55 eV peak after MG adsorption is likely due to the surface sensitivity of XPS, which detects only the topmost 10 nm. On the other hand, with a pore diameter of 14.85 nm, our prepared mesoporous composite allows dye molecules to adsorb deeper, which is beyond XPS detection. These may also explain the absence of a peak at 398.55 eV. Moreover, the atomic percentage of N 1s decreased from 4.24% to 1.19% after MG adsorption (Table S1†). The decrease in N 1s intensity after adsorption can be explained by the charge transfer mechanism between the nitrogen atoms in Malachite Green and the metals in the composite (*i.e.* Ce Ag, Co, Fe). The nitrogen atoms in the dimethylamino groups of Malachite Green possess lone pairs of electrons, which can act as electron donors. Meanwhile, Ce^4+^, with its high oxidation state and available empty 4f orbitals, can serve as an electron acceptor. This creates a charge transfer interaction, where nitrogen donates electron density to the empty 4f orbital of cerium. From the XPS spectrum of Ce 3d (Fig. 4a), it is observed that there are six distinctive peaks of Ce 3d divided into two groups of V and U types, linking to the two states Ce 3d_5/2_ and Ce 3d_3/2_.^74^ Before adsorption, the peaks denoted as *V* (882.63 eV), *V*″ (898.45 eV), *U* (900.34 eV), and *U*″ (916.44 eV) were attributed to Ce^4+^ and *u*′ (904.32) and *v*′ (885.43) for Ce^3+^.^75,76^ These peaks confirm that in the composite, both cerium Ce^3+^ and Ce^4+^ states are present. After adsorption, the Ce^4+^ binding energy shifted from 882.63 to 881.76 eV, 898.45 to 898.47 eV, 900.34 to 900.98 eV, and 916.44 to 916.24 eV, whereas the Ce^3+^ binding energy shifted from 885.46 to 884.27 eV and 904.32 to 904.24 eV (Fig. 4b). This shifting indicated the changes in the electronic environment around cerium, likely due to the co-ordination or charge transfer from the nitrogen loan pair of MG, which influences the reduction in the N 1s signal after adsorption. Moreover, the XPS peaks of Ag 3d appeared for the Ag 3d_5/2_ and Ag 3d_3/2_ states of Ag at 367.78 eV and 373.77 eV (Fig. 4c), respectively, confirming the doping of Ag into the composite.^77^ For silver (Ag), the peaks corresponding to the Ag 3d_5/2_ and Ag 3d_3/2_ states shift from 367.78 eV and 373.77 eV before adsorption to 367.97 eV and 373.98 eV after adsorption (Fig. 4d). The doublet peak at binding energies of 780.33 eV and 795 eV (Fig. 4e) represent Co 2p_3/2_ and Co 2p_1/2_ electronic states, respectively, of the Co 2p XPS spectra.^78^ For cobalt (Co), the Co 2p_3/2_ and Co 2p_1/2_ peaks shift from 780.33 eV and 795.00 eV before adsorption to 780.76 eV and 795.88 eV after adsorption (Fig. 4f), indicating the interaction between the MG molecules and the cobalt ions, which may involve electron donation from the dye to the metal centre. The Fe 2p XPS spectrum of the composite before adsorption reveals two primary peaks at 710.2 eV and 723.39 eV (Fig. 4g), corresponding to Fe 2p_3/2_ and Fe 2p_1/2_, respectively, which are characteristic of Fe^3+^ in ferrite materials.^79,80^ The satellite peak observed at approximately 718 eV further confirms the presence of Fe^3+^.^81^ After adsorption of MG, the iron (Fe) peaks for Fe 2p_3/2_ and Fe 2p_1/2_ shift from 710.2 eV and 723.39 eV to 710.29 eV and 723.59 eV (Fig. 4h). These shifts in the Fe 2p_3/2_ and 2p_1/2_ peaks suggests that there is no detectable reduction of Fe^3+^ to Fe^2+^ after dye adsorption, implying that Fe does not undergo redox changes during the adsorption process. This stability of the Fe oxidation states highlights the role of surface adsorption mechanisms rather than redox interactions between Fe and the dye. Overall, all shifts indicate a possible coordination interaction between the nitrogen atoms of MG and metal ions in the composite, suggesting that an inner sphere surface complexation occurs during the adsorption process.

### 3.3 Adsorption mechanism

In the composite, cellulose acetate (CA), polyvinylpyrrolidone (PVP), cerium oxide (CeO_2_), and Ag-doped cobalt ferrite interacted through coordination, hydrogen bonding, and electrostatic effects. The hydroxyl (–OH) and carbonyl (C=O) groups in CA formed hydrogen bonds with CeO_2_ and PVP and coordinated with metal ions in the ferrite. PVP contributed additional stability by coordinating through its nitrogen atoms with Ce and Ag ions and forming hydrogen bonds with CA's hydroxyl groups. CeO_2_, with its surface oxygen and Ce^3+^/Ce^4+^ ions, participates in both hydrogen bonding with the hydroxyl (–OH) group of CA and coordination with carbonyl groups in CA and PVP. Cerium in the composite also allowed possible electron transfer interactions with the N lone pair of PVP. Hence, it is observed from the XPS spectra of cerium that both Ce^3+^ and Ce^4+^ states are present before adsorption. Ag-doped cobalt ferrite further stabilized the composite through coordination between metal ions with the oxygen of CA and oxygen as well as nitrogen of PVP. Additionally, the Ag ions provided electrostatic interactions with the nitrogen atoms in PVP, contributing to the overall stability of the composite. These combined interactions create a cohesive, stabilized network within the composite structure.

Adsorption is a surface phenomenon where adsorbates (ions or molecules) are held together by either van der Waals forces (physisorption) or chemical bonds (chemisorption). In this study, the adsorption of Malachite Green dye onto the surface of the ACFCeP composite can be attributed primarily to five main factors. (i) electrostatic interaction, (ii) H-bonding, (iii) surface complexation, (iv) pi–pi interaction, and (v) pore filling. (i) Electrostatic interactions: the electrostatic attraction between the positively charged Malachite Green (MG) molecules and the negatively charged functional groups (such as –OH groups in cellulose acetate and carbonyl groups in both PVP and cellulose acetate) plays a significant role in adsorption. These functional groups act as active binding sites for MG molecules.^82^ (ii) Hydrogen bonding: hydrogen bonding is another important interaction between the nitrogen-containing groups (dimethylamino groups) of MG and the hydrogen of the –OH group of cellulose acetate. This interaction enhances the adsorption process. (iii) Surface complexation and charge transfer: H. Zhu *et al.* reported that in surface complexation, the electron pair donor and electron acceptor interact to form various complexes.^83^ The XPS analysis reveals significant shifts in the binding energies of several metal ions before and after the adsorption of Malachite Green (MG), which supports the involvement of surface complexation and charge transfer interactions in the adsorption mechanism. This shift indicates a possible coordination interaction between the nitrogen atoms of MG and metal ions in the composite, suggesting that inner sphere surface complexation occurs during the adsorption process and the hypothesis of metal–dye complex formation (iv) π–π interactions: π–π stacking occurs between the aromatic rings of MG and the planar surfaces of cellulose acetate and PVP.^84^ These non-covalent interactions stabilize the dye molecules on the adsorbent surface.

**Scheme 2:**
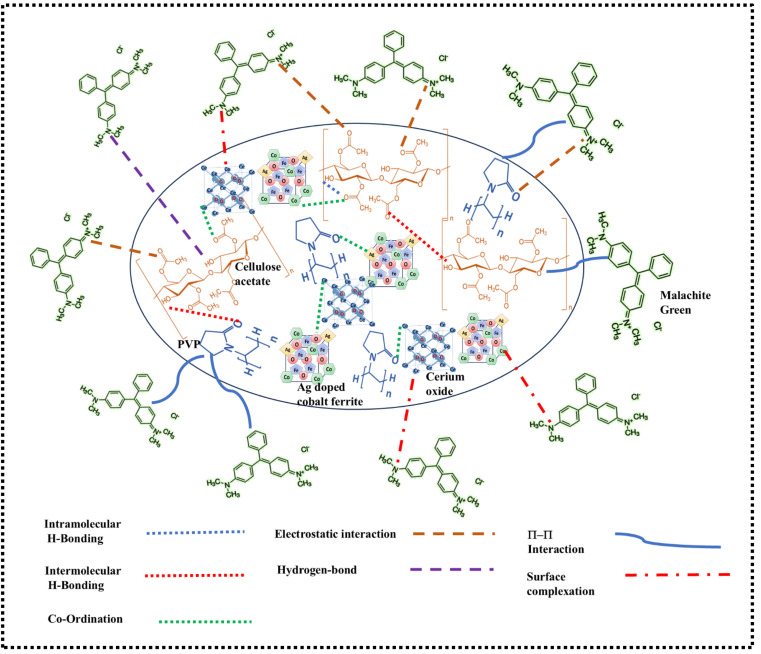
Adsorption mechanism of the Malachite green dye on ACFCeP composite.

Similar interactions were reported by A. A. Alqadami *et al.* where they observed electrostatic interactions, π–π stacking, and hydrogen bonding in the adsorption of Malachite Green onto Fe_3_O_4_@AMCA-MIL53(Al) composites.^85^ (v) Pore filling: the micropores in the ACFCeP composite structure may also contribute to the adsorption of MG by pore filling, where dye molecules are physically trapped within the porous network. From the BJH pore volume analysis, it is found that the composite possesses a pore volume of 0.0216 cm^3^ g^−1^, which also contributes to the dye adsorption mechanism. Scheme 2 presents the adsorption mechanism of the dye with the film.

Notably, no catalytic effect of MG on the ACFCeP film was observed in our experiments, as no reaction products indicative of catalytic degradation or chemical transformation of MG were detected in our analysis.

### 3.4 Morphological analysis

**Fig. 5:**
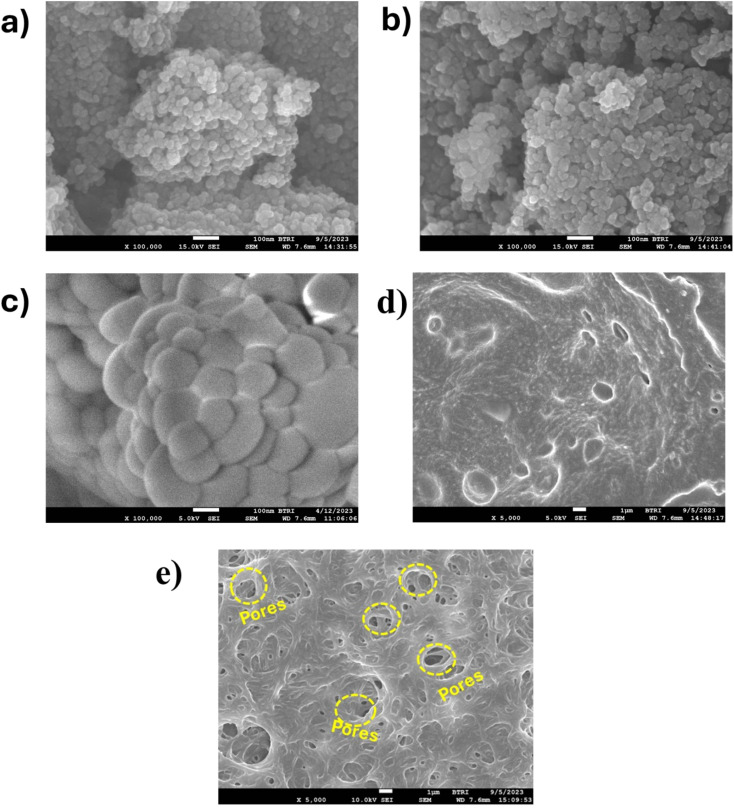
FE-SEM image of cobalt ferrite (a); Ag-doped cobalt ferrite (b); cerium oxide (c); CA/PVP polymer blend (d); ACFCeP composite (e).

FE-SEM was used to analyze and observe the surface morphology of the samples. In Fig. 5a and b, the images of CF and ACF, respectively, show the sample possesses a very dense arrangement of uniformly distributed spherical-shaped nanoparticles. Also, both cobalt ferrite (CF) and Ag-doped cobalt ferrite (ACF) nanoparticles are visibly present in numerous agglomeration forms. The agglomeration of spherical particles observed in the SEM images can be attributed to van der Waals forces and particle–particle interactions, which are common in nanoscale materials.^86^Fig. 5c illustrates the spherical CeO_2_ particles.

Comparing the FE-SEM image of CA/PVP in Fig. 5d and ACFCeP in Fig. 5e, many pores and cavities can be observed. The mixing of nanoparticles (ACF and CeO_2_) with the polymeric matrix (CA/PVP), along with the solvent evaporation process, likely contributed to the formation of pores and cavities within the composite structure.^87,88^ During solvent evaporation, voids can be formed as the solvent leaves the system, especially in the presence of nanoparticles, which can disturb the packing of the polymer chains and increase the porosity.

The EDX analysis of CF and ACF are shown in Fig. S1a and b.† Comparing the atomic percentages of CF and ACF given in Tables S3 and S4,† respectively, the decreased atom percentage of Co indicates that Co was mostly replaced by Ag in ACF.

### 3.5 Brunauer–Emmett–Teller (BET) specific surface area analysis

**Fig. 6:**
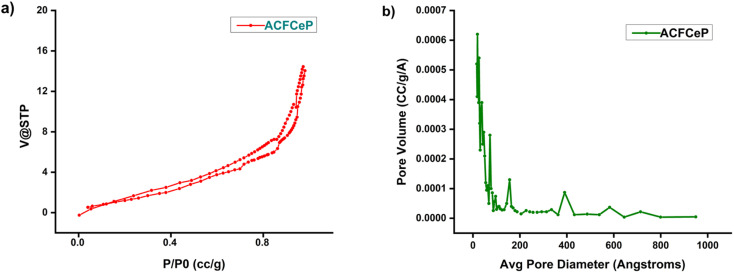
N_2_ gas adsorption–desorption isotherm (a); BJH adsorption pore size distribution (b).

The surface area and porosity of an adsorbent have a significant effect on adsorption performance.^89,90^ The N_2_ gas adsorption and desorption isotherms of the prepared ACFCeP composite film are depicted in Fig. 6a and 4b.

Table 1 shows the obtained BET surface area, pore size diameter and pore volume of the ACFCeP film. IUPAC (International Union of Pure and Applied Chemistry) classification was followed to determine the isotherms and pores of the film. According to IUPAC, pores with a diameter smaller than 2 nm are defined as micropores, mesopores with a diameter of 2–50 nm and macropores with a diameter greater than 50 nm.^91^ The BET analysis shows that the composite film exhibits a Type IV isotherm with an H4 type hysteresis loop.^92^ The specific surface area of the ACFCeP composite shows a moderate surface area of 5.824 m^2^ g^−1^, which is beneficial for the adsorption of organic dye molecules. The composite's pore volume is 0.0216 cm^3^ g^−1^, while the average pore diameter of 14.85 nm indicates the composite's mesoporous structure, which contributes to the diffusion of dye molecules into the composite film. Importantly, the molecular size of Malachite Green is 0.82 nm.^93^ Hence, it can fit into the pores of the ACFCeP composite during adsorption and can make the ACFCeP composite a suitable candidate for adsorption applications. Although the surface area is not particularly high, research by Chaukura *et al.* and N. El Badawi *et al.* shows that porous materials with similar surface areas can perform well in dye adsorption due to the accessibility of pores.^94,95^

**Table 1:** BET surface area, pore volume, and pore size diameter of the ACFCeP composite film

Specific surface area (m^2^ g^−1^)	5.824
Pore volume BJH (cm^3^ g^−1^)	0.0216
Average pore diameter (nm)	14.85 (mesopores)

### 3.6 Vibrating sample magnetometer (VSM) analysis

**Fig. 7:**
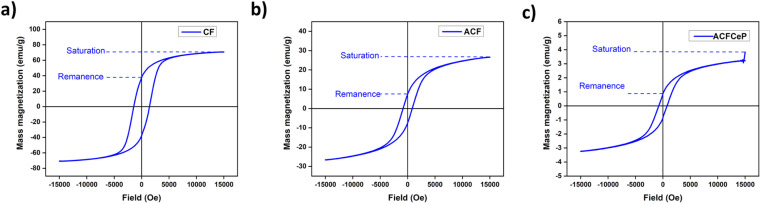
Magnetic hysteresis (M−H) curve of cobalt ferrite (CF) (a); Ag-doped cobalt ferrite (ACF) (b) and ACFCeP composite (c).

The magnetic properties of the CF, ACF and ACFCeP composite film were examined using VSM analysis. The sample's magnetic hysteresis (M−H) curves obtained at room temperature are shown in Fig. 7a–c.

Fig. 7 indicates the saturation magnetization (Ms) and remnant magnetization (Mr) of cobalt ferrite to be 82.9858 emu g^−1^ and 37.6368 emu g^−1^. A big hysteresis loop obtained from the M−H curve of cobalt ferrite (CF) suggests that it has ferrimagnetism.^96^ After silver doping, the hysteresis loop of Ag-doped cobalt ferrite (ACF) shows lower saturation magnetization and remanent magnetization, which are 29.8742 emu g^−1^ and 7.4171 emu g^−1^, respectively. The final ACFCeP composite exhibits a Ms of 3.7123 emu g^−1^ and Mr of 0.8467 emu g^−1^. Though ACFCeP shows lower magnetization values compared to pure cobalt ferrite, it also gives a visible hysteresis loop in the M−H curve, indicating the presence of magnetic properties in it.

### 3.7 Adsorption study of Malachite Green dye

#### 3.7.1 Comparison of MG dye removal efficiency of CF, ACF, CA/PVP and ACFCeP

The removal percentage of the MG dye was evaluated using cobalt ferrite nanoparticles, Ag-doped cobalt ferrite nanoparticles, CA/PVP polymer blend film and ACFCeP composite film individually as an adsorbent. 50 mg of each adsorbent was taken separately in four 30 mL, 50 ppm aqueous MG dye solutions at pH 7. The adsorption experiment was conducted in a shaker at 200 rpm for 90 min. UV-vis spectrometry was used to measure the absorbance of the residual solutions and determine the equilibrium concentration with the help of the calibration curve. The result given in Fig. S2† illustrates that cobalt ferrite nanoparticles show 78% removal of MG dye, whereas in Ag-doped cobalt ferrite (ACF), the removal percentage increased to 86%. Generally, cobalt ferrite has a high surface area as a nanomaterial, and perhaps after doping with Ag, the surface area became higher with more active spaces in the structure to entrap more dye molecules.^53^ Moreover, XPS analysis supports that the enhanced dye removal is also due to the metal–dye interactions *via* coordination. The XPS binding energy shifts for Ag, Co, and Fe after MG adsorption, confirming metal–dye coordination interactions, which indicates that after MG adsorption, there is a coordination interaction of Ag with the dye along with Co and Fe, and hence, the removal% increase in ACF is comparable to CF. There was also an increase in the removal percentage of the CA/PVP film from 70 to 93% after AC and CeO_2_ were added to it. These additional particles increased the surface area and created more voids on the composite surface, which enhanced the removal efficiency of the final composite film, leading to high MG dye removal. Additionally, from the mechanism of the dye removal analysis, it was found that in the CA/PVP film, only the electrostatic interaction, H-bond and π–π stacking between the aromatic rings in Malachite Green and the planar surfaces of PVP and cellulose acetate are responsible for adsorption and hence the MG dye removal. On the other hand, in the final composite, along with this electrostatic interaction, the H-bond and π–π stacking mechanisms, coordination and charge transfer between nitrogen atoms in MG and metal atoms (Ag, Co, Fe, Ce) of the composite had occurred. Therefore, the removal percentage increased in ACFCeP than in CA/PVP.

#### 3.7.2 Influence of pH and adsorbent dosage

Solution pH is one of the most significant factors as it affects both ionization and adsorption of dyes on adsorbent's active sites.^97^ The adsorption process is influenced by a change in pH, due to which the functional groups present on the adsorbate and adsorbent can dissociate.^98^ Another important factor is the point of zero charge (pH_pzc_) at which an adsorbent shows zero surface charge. If the pH is above pH_pzc_, the adsorbent surface is negatively charged so that cations can be adsorbed. Then again, if pH is below its pH_pzc,_ the surface of the adsorbent is positively charged.

**Fig. 8:**
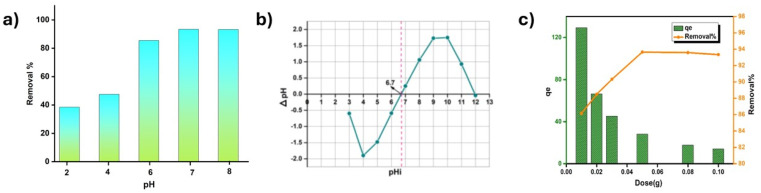
Effect of solution pH on Malachite Green dye adsorption (a); point of zero charge of the ACFCeP composite (b); effect of adsorbent dosage on the adsorption of Malachite Green (c) (pH = 2–8, dose = 50 mg, time = 90 min).

To understand the relation between solution pH and MG dye adsorption process on ACFCeP, 50 mg of the composite was taken in a 50 ppm dye solution with pH ranging from 2 to 8, maintaining a contact time of 90 min. The result (Fig. 8a) shows that cationic MG dye adsorption is trivial in an acidic solution (pH 2). This happens as the H^+^ ion is present in high concentration, which replaces the MG dye molecule during adsorption. With increasing pH, the concentration of H^+^ ion decreases, and the removal percentage of the cationic MG dye increases. At pH 7, the removal% reaches the maximum at 93.3%. Fig. 8b shows that the ACFCeP composite film has a pH_pzc_ of 6.7, which is below pH 7, indicating that the adsorbent surface is negatively charged and able to bind with the cationic MG dye.

Another important parameter is adsorbent dosage, which fixes the adsorbent's capacity for a certain initial dye concentration. For the batch experiment, ACFCeP adsorbents of weight 10–100 mg were taken separately in 30 mL of 50 ppm MG dye solutions, and the shaking speed was maintained at 200 rpm for 90 min.

The graph in Fig. 8c depicts that the adsorbent capacity of the ACFCeP film decreases with increasing adsorbent dosage. These may result from the aggregation or overlapping of adsorption sites and increased diffusion pathways.^67^ It is also visible that the removal percentage improved with the increasing quantity of adsorbents. The reason might be the accessibility to more active sites and the rise in surface area with the increasing adsorbent molecule.^99^ From the experiment, it is observed that the optimum adsorbent dose is 50 mg as it shows a maximum removal percentage of 94% with *q*_e_ of 110 mg g^−1^, and further addition of the adsorbent does not influence the obtained values.

#### 3.7.3 Influence of the initial concentration of MG and adsorption isotherm

**Fig. 9:**
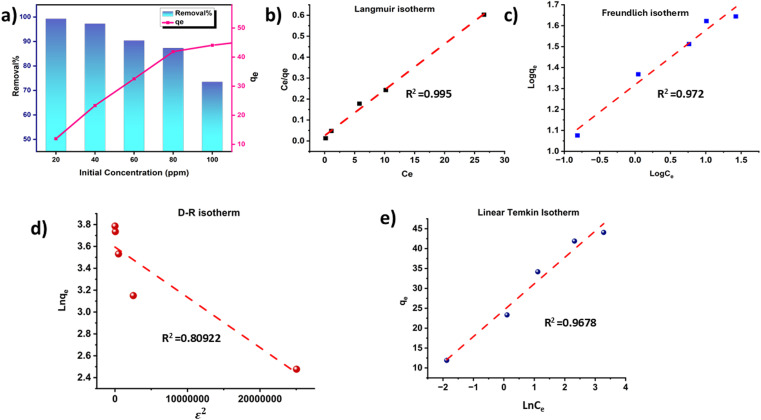
Influence of initial concentration on the adsorption of MG dye (a); linear fit Langmuir isotherm (b); linear fit Freundlich isotherm (c); linear fit D–R isotherm (d) and linear fit Temkin isotherm (e) for the adsorption of Malachite Green (pH = 7, dose = 50 mg, time = 90 min).

To study the influence of the initial concentration on the adsorption process, different concentrations of the dye solution varying from 20–100 ppm were prepared. 50 mg of adsorbents were added into a 30 mL solution of the dye at pH 7, and the contact time was maintained at 90 min. The result is illustrated in Fig. 9a.

With increasing initial dye concentration, the percentage removal of the dye decreased but the adsorption capacity increased. This tendency indicates that a certain quantity of the adsorbent has almost a fixed amount of adsorption sites. So, the adsorbent cannot absorb dye molecules after the adsorption sites are occupied.^100^ Therefore, at lower initial dye concentrations, the composite adsorbent can adsorb the highest number of dye molecules. On the contrary, at higher initial dye concentrations, the active sites of the composite become saturated and the dye removal percentage decreases.

**Fig. 10:**
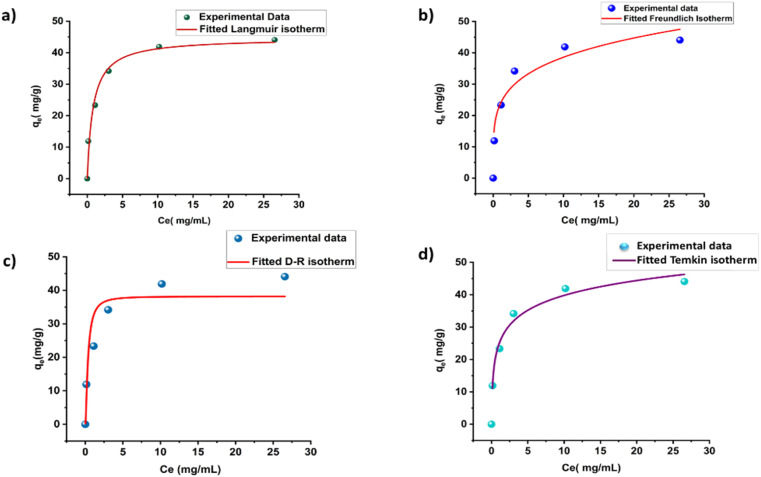
Non-linear fit Langmuir isotherm (a); non-linear fit Freundlich isotherm (b); non-linear fit D–R isotherm (c) and non-linear fit Temkin isotherm (d) for the adsorption of Malachite Green (pH = 7, dose = 50 mg, time = 90 min).

The relation between the adsorbate MG dye molecules and the ACFCeP surface is examined by the Langmuir, Freundlich, Dubinin–Radushkevich and Temkin isotherms, which are shown in Fig. 9b–e, respectively, in their linear forms. The non-linear forms of the corresponding isotherms are also demonstrated in Fig. 10a–d.

*K* and *q*_max_ values are measured from the slope and intercept of the linear plots of the Langmuir and D-R isotherms, respectively, using eqn (21) and (22). *q*_max_ is the theoretical maximum adsorption capacity, which is the highest adsorbate quantity that can be adsorbed per unit mass of the adsorbent (mg g^−1^).^101^ The equation shows a *q*_max_ value of 45.66 mg g^−1^ and 36.39 mg g^−1^ for Langmuir and D-R linear models, respectively.

21 *y* = 0.0219*x* + 0.0252

22 *y* = −0.00000005*x* + 3.5944

23 *y* = 0.2606*x* + 1.318

24 *y* = 6.633*x* + 24.514

The linear form of the Freundlich isotherm for the composite film is expressed by eqn (23), which gives the values of *K*_f_ and 1/*n*. As *n* is a function of the strength of adsorption, adsorption becomes more favorable with the increasing value of *n*.^102^ The value of *n* for good adsorption is between 2–10; for difficult adsorption, it is 1–2 and less than 1 for very poor adsorption.^103^ The value of *n* found in this study is 3.8 at room temperature, which indicates that adsorption is satisfactory in this case. The *R*^2^ value is found to be 0.972, which represents the moderate following of the Freundlich isotherm. The linear Temkin isotherm equation is shown in eqn (24). From eqn (11) and Table 2, the sign of *b* is positive, which indicates the process is endothermic.^104^ The data of all the non-linear isotherms are provided in Table S5.† The best-fitted isotherm model is indicated by the maximum value of the regression factor (*R*^2^). Among the linear and non-linear isotherms, the D–R isotherm was proven the most unfitted in both forms, with the lowest *R*^2^ values. Among all linear and non-linear models, both forms of the Langmuir isotherm and linear form of the Freundlich isotherm exhibited *R*^2^ values of 0.99 and 0.97, which is close to 1. Therefore, it can be said that the ACFCeP composite followed the linear Langmuir and Freundlich isotherms than the other isotherms. Furthermore, the adsorption capacity is also influenced by the pore volume, which can be observed from the calculation based on a pore volume of 0.0216 cm^3^ g^−1^ and the density of MG (1.2 g cm^−3^), suggesting an expected adsorption capacity of approximately 26 mg g^−1^. This calculation underscores the significance of pore volume in the adsorption process, as it directly impacts the capacity values.

**Table 2:** Linear isotherm rate constant and regression coefficient values for Langmuir, Freundlich, D–R and Temkin isotherms

Isotherm model	Parameter	Value for ACFCeP composite
Langmuir	*q*_max_ (mg g^−1^)	45.66
	*K* (L mg^−1^)	0.869
	*R* ^2^	0.9956
Freundlich	*K*_f_ (mg g^−1^)	20.79
	*n*	3.8
	*R* ^2^	0.972
D–R	*β*_DR_	4.60 × 10^−8^
	*q*_max_ (mg g^−1^)	36.3924014
	*R* ^2^	0.80922
Temkin	*A* (unitless)	6.63
	*K*_T_ (L mg^−1^)	40.3
	*b* (kJ mol^−1^)	373.69
	*R* ^2^	0.9678

#### 3.7.4 Influence of contact time and adsorption kinetics study

A fast adsorption period is highly expected in wastewater treatment for quick dye adsorption and equilibrium establishment.^105^ This is a significant factor affecting the efficiency of adsorbents. To study the contact time on MG dye, 30 mL dye solutions of 20, 60 and 100 ppm were prepared in which 50 mg adsorbents were added. The dye solutions were positioned in a reciprocating shaker operating at 200 rpm, and the pH was maintained at 7.

**Fig. 11:**
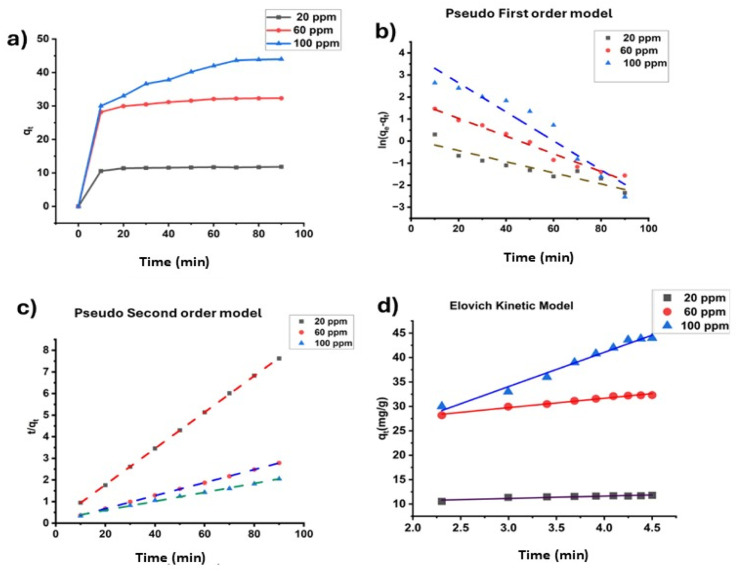
Relation between the contact time and the adsorption process of Malachite Green (a); linear fitting of pseudo first order (b); linear fitting of pseudo second order (c) and linear fitting of the Elovich kinetic model (d) for the adsorption of Malachite Green on ACFCeP composite (pH = 7, dose = 50 mg, time = 90 min).

The relation between the contact time and adsorption capacity is shown in Fig. 11a for the three different concentrations. From the graph, it is visible that adsorption capacity rises rapidly in the first 10 min for all studied concentrations. Then, adsorption capacity becomes constant after 50 min for 20, 60 ppm and 70 min for 100 ppm. The plot suggests that with the increase in time, open sites for adsorption on the ACFCeP surface are decreased and at the plateau, the adsorption efficiency does not change anymore with any increase in adsorption time.^106^

**Fig. 12:**
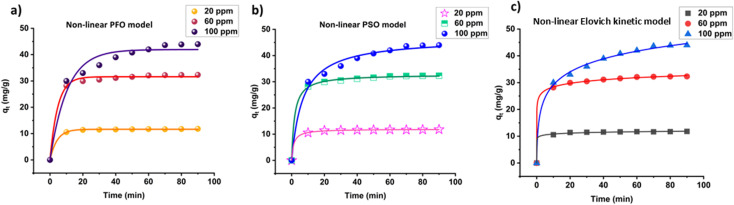
Non-linear fitting of pseudo first order (a); non-linear fitting of pseudo second order (b) and non-linear fitting of Elovich (c) kinetic model for the adsorption of Malachite Green on ACFCeP composite (pH = 7, dose = 50 mg, time = 90 min).

To study the ACFCeP adsorption kinetics, MG dye solutions of different concentrations (20, 60, 100 ppm) are taken. The linear pseudo first order, pseudo second order and Elovich plots are shown in Fig. 11b–d, and the non-linear plots are demonstrated in Fig. 12a–c.

The adsorbent's adsorption capacity at equilibrium and rate constant are determined from the slope and intercept of each linear plot, respectively, and displayed in Table 3. The data for the non-linear plots are presented in Table S6.† The linear first order, second order and Elovich equations for three specific concentrations are displayed in Table S7.†

**Table 3:** Linear pseudo first order, pseudo second order and Elovich kinetic model parameters

Kinetic model and parameter	20 ppm	60 ppm	100 ppm
Experimental *q*_e_ (mg g^−1^)	11.9081	32.5260	44.0642

**Pseudo first order (PFO)**			
*q*_e_ (mg g^−1^)	1.0796	6.2651	50.3702
*K*_1_ (min^−1^)	0.0252	0.0402	0.0658
*R* ^2^	0.8989	0.9794	0.9356

**Pseudo second order (PSO)**			
*q*_e_ (mg g^−1^)	11.9047	33.1125	48.3091
*K*_2_ (g mg^−1^ min^−1^)	0.0728	0.0137	0.0019
*R* ^2^	0.9999	0.9991	0.9914

**Elovich kinetics model**			
*α* (mg g^−1^ min^−1^)	0.142450142	0.526316	2.083333
*β* (g mg^−1^)	44.6014365	551 722.9	2.87000000
*R* ^2^	0.815	0.979	0.979

Comparing the *R*^2^ values, it is noticeable that pseudo second order kinetics and Elovich model both showed satisfactory *R*^2^ values in the non-linear forms but only the pseudo second order kinetic reached an *R*^2^ value close to 1 in the linear form for three different concentrations. Hence, it is suggested that the linear pseudo second order kinetics is more suitable than any other kinetic models for the adsorption of MG dye on the ACCFCeP film.

#### 3.7.5 Reusability of the ACFCeP composite

Adsorbents must be efficient as well as recyclable to treat industrial effluents. The treatment process becomes economically feasible when the adsorbent can be simply regenerated after each cycle and stays useable for a long time. In this study, the recyclability of the ACFCeP composite was examined up to three cycles in 50 ppm dye solutions for 90 min. The pH was maintained at 7. The ACFCeP composite was washed with HCl to eliminate the adsorbed dye at the termination of each cycle. To neutralize the pH, washing and drying of the film was done before the next cycle.

**Fig. 13:**
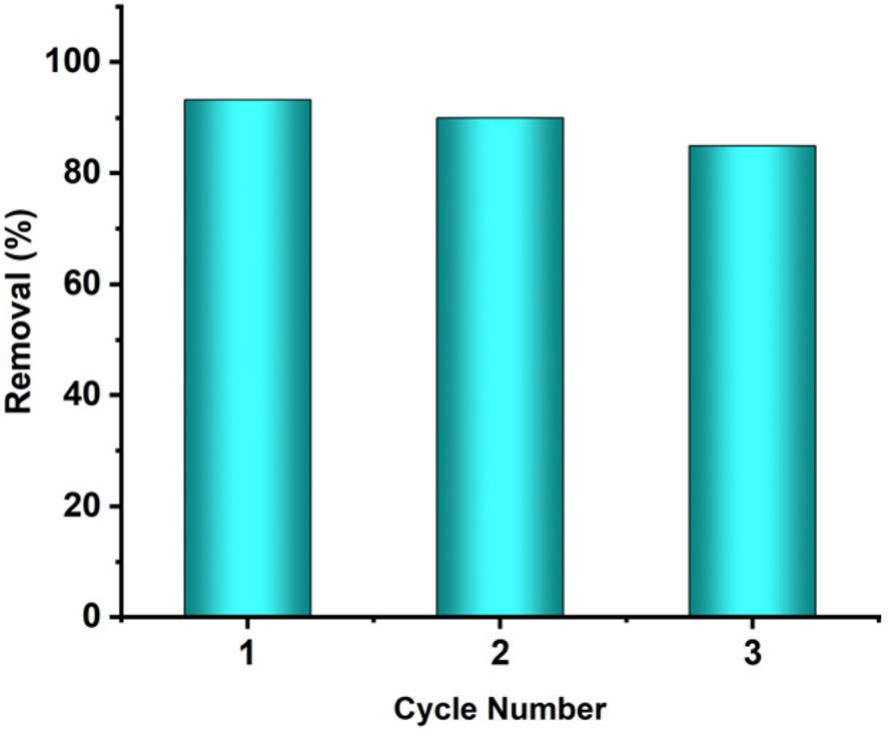
Demonstration of the reusability of the ACFCeP composite for removing MG up to three cycles.

Fig. 13 shows that the dye removal percentage drops from 93.3% in the 1st cycle to 85% in the 3rd cycle. The removal percentage reduced gradually due to the partial desorption of dye molecules when regenerated after each cycle. Besides, blocking the pores by the dye molecules also contributes to a successive decrease in performance.^107,108^ So, experimentally, it is evident that the ACFCeP composite film has the potential for long-term reusability in industrial applications for removing organic dye molecules. Table 4 compares the adsorption capacities of the MG dye on various polymers and nanoparticle composites with the newly developed magnetic composite.

**Table 4:** Comparison of the maximum adsorption capacities for MG dye reported in different studies

Adsorbent	*Q*_max_ (mg g^−1^)	References
Chitin hydrogel	33.57	109
Graphene oxide (GO)-cellulose bead (GOCB)	17.86	110
Activated carbon-chitosan-SDS film	4.80	8
Zinc oxide-chitosan composite	11.00	111
ZnFe_2_O_4_-polyaniline-graphene oxide nanocomposite	9.17	112
Zeolite loaded PVA-CMC-sodium alginate membrane	29.58	113
Mn–Fe layered double hydroxides-polyethersulfone (PES) membrane	13.49	114
Magnetic activated carbon	36.36	115
Ag_0.2_Co_0.8_ Fe_2_O_4_/CeO_2_/cellulose acetate/polyvinylpyrrolidone (ACFCeP) composite film	45.66	This work
Magnetic nano copper ferrite, CuFe_2_O_4_	22	116
Bentonite	7.72	117
Sugarcane dust	4.88	118

## 4 Conclusions

In conclusion, this study introduces a straightforward synthesis method for an Ag_0.2_Co_0.8_Fe_2_O_4_/CeO_2_/CA/PVP (ACFCeP) composite film aimed at removing hazardous Malachite Green dye from industrial effluents. Through XPS analysis, it was revealed that the adsorption mechanism involves surface complexation *via* coordination of the metal present (Ag, Co, Fe, Ce) in the composite with the MG dye molecule. Also, other features like hydrogen bonding, electrostatic and π–π interactions are also responsible for adsorption as directed by changes in bond intensity. Moreover, the 14.8 nm mesoporous structure of the composite facilitates efficient dye removal by trapping the molecules within its pores. Additionally, the adsorption isotherm exhibited linear regression coefficients (*R*^2^) of 0.99 for the Langmuir model and 0.97 for the Freundlich model. Furthermore, batch adsorption experiments demonstrated a maximum adsorption capacity of 45.66 mg g^−1^ at pH 7. Notably, kinetic studies followed a pseudo-second-order model (*R*^2^ > 0.99), with equilibrium achieved within 50 min for dye concentrations of 20 and 60 ppm and within 70 min for 100 ppm. Importantly, the composite maintained an 85% dye removal efficiency after three cycles, highlighting its excellent reusability. Additionally, the composite's saturation magnetization of 3.7123 emu g^−1^ and strong hysteresis loop enable magnetic removal post-treatment. Consequently, these findings suggest that the synthesized composite film holds significant potential for real-world applications, particularly for the adsorption purpose of cationic dyes from aqueous solutions and industrial waste effluents.

## Data availability

The data supporting this article are provided within the main article and the accompanying ESI.†

## Author contributions

Monika Mahmud designed the study, conducted the experiments, wrote the manuscript, and supervised the entire research. Nafisa Tabassum conducted the experiments. Raamisa Anjum contributed to writing the manuscript. Papia Haque and Samina Ahmed also contributed to the supervision of the research. Md. Sahadat Hossain was responsible for the XRD phase identification analysis. Mashrafi Bin Mobarak performed the functional group analysis. Md. Saiful Quddus conducted the surface area and porosity analysis, as well as the XPS analysis. Lutfor Rahman conducted the VSM analysis. Fariha Chowdhury and Dipa Islam carried out the morphological investigation experiments.

## Conflicts of interest

There is no conflict of interest to report.

## Supplementary Material

RA-014-D4RA06315E-s001
