# Supplementary material for: Ag–Co ferrite-based magnetic polymeric composite film: a breakthrough in cationic dye remediation for sustainable environment
Source: RSC Adv. 2024 Nov 15;14(49):36557–75. doi: 10.1039/d4ra06315e (PMC11565276; doi:10.1039/d4ra06315e)
Supplement: RA-014-D4RA06315E-s001 [file RA-014-D4RA06315E-s001.pdf]

Supplementary Information (SI) for RSC Advances.
This journal is © The Royal Society of Chemistry 2024

Supporting Information

Table S1: Atomic % of composite element before and after MG adsorption

Atom	Before MG adsorption (%)	After MG adsorption (%)
C 1s	63.7	62.32
O 1s	25.5	29
N 1S	4.24	1.19
Ag 3d	0.56	0.6
Co 2p	1.27	1.55
Fe 2p	3.66	4.56
Ce	1.07	0.78

Table S2: XPS before and after MG adsorption bond energy and intensity

	Before MG adsorption		After MG adsorption	
**Bond Name**	**Binding energy**	**Intensity (CPS)**	**Binding energy**	**Intensity (CPS)**
C1s Scan A (C-C)	284.86	40106.3	284.83	42252.3
C1s Scan B (O-C-O)	286.27	15854.41	286.64	17677
C1s Scan C (O-C=O)	288.35	7441.29	288.92	8454.88
O1s Scan A (O-H)	532.23	32148.51	532.63	41113.02
O1s Scan B (O=C)	529.34	18181.97	529.65	20103.88
N1s Scan A (N-O)	399.92	5814.28	399.86	1215.2
N1s Scan B (C-N/C=N)	398.55	1107.26	----	------
N1s Scan C (Bond related to C-N)	404.58	750.63	404.95	599.57
Ag3d5 Scan A	367.82	13748.48	367.97	12876.24
Co2p	780.33	3555.78	780.76	4189.77
Fe2p	710.2	7602.87	710.29	9940.87
Ce3d	882.63	4342.9	884.27	2745.11

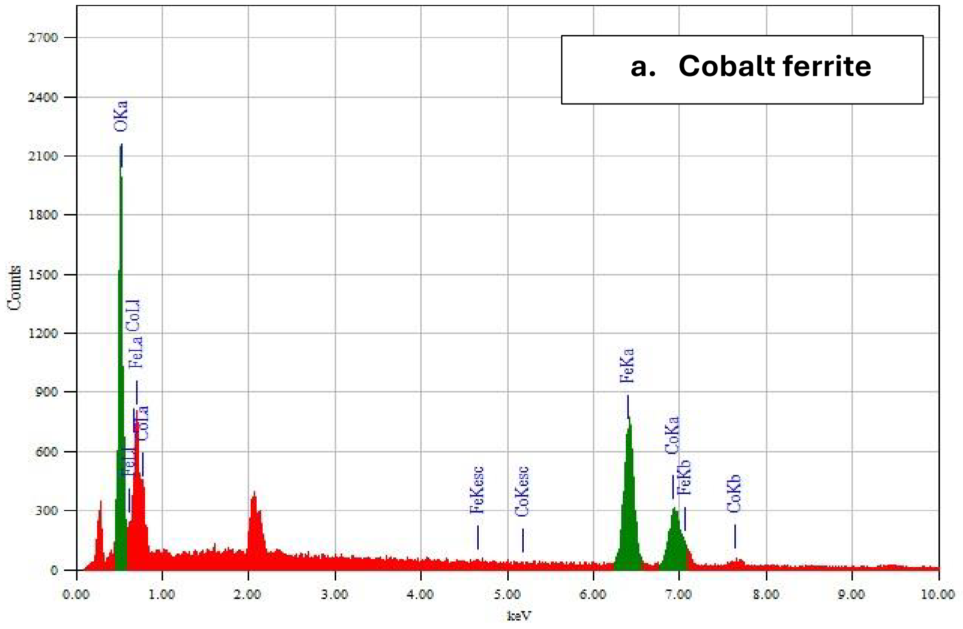

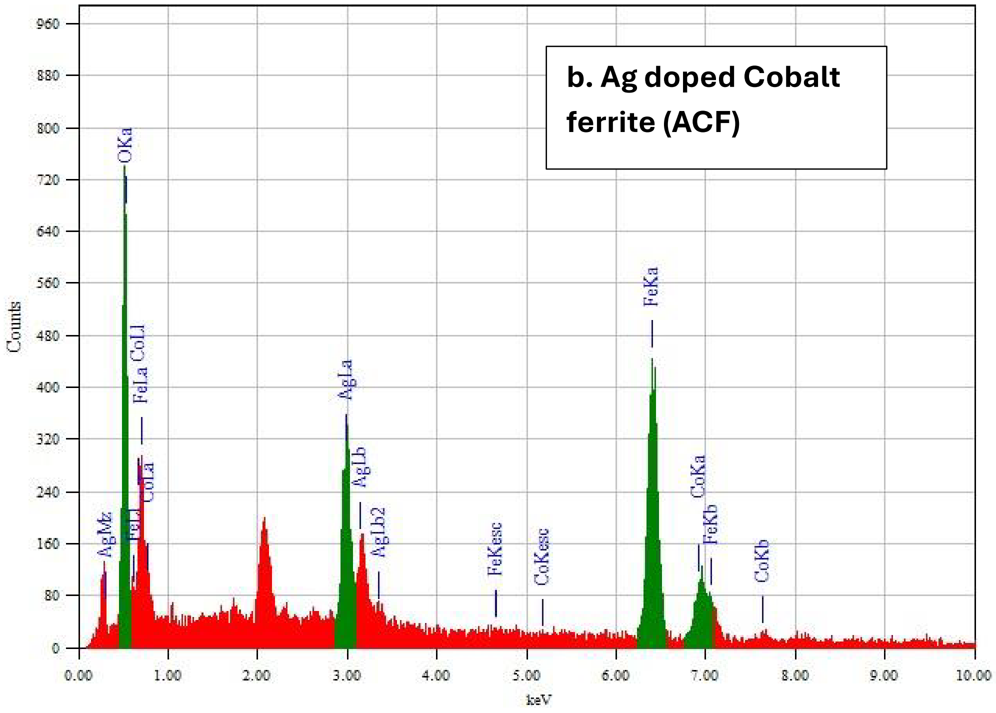

Figure S1: EDX analysis of a. CF, b. ACF

Table-S3: Elemental analysis of CF

Element	Mass%	Atom%
Oxygen	25.33	54.65
Iron	49.93	30.86
Cobalt	24.74	14.49

Table-S4: Elemental analysis of ACF

Element	Mass%	Atom%
Oxygen	21.90	52.67
Iron	47.65	32.83
Cobalt	12.25	8.00
Silver	18.20	6.49

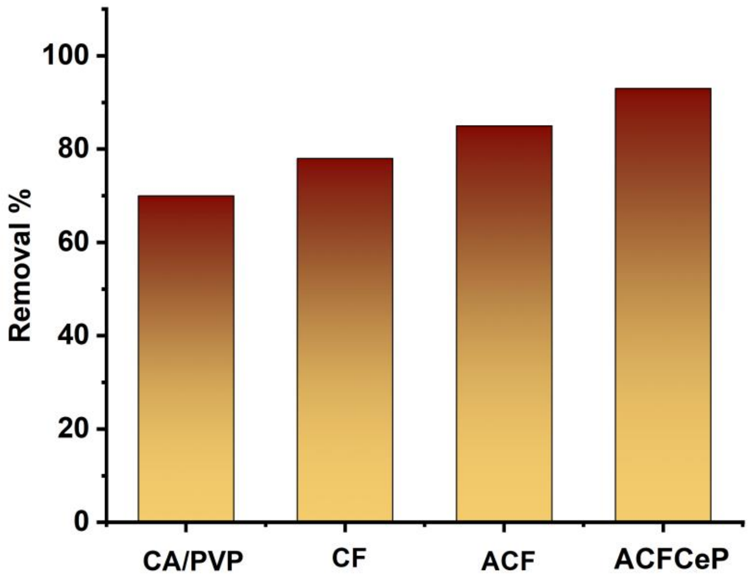

Figure-S2: Comparison of MG dye removal efficiency of CF, ACF, CA/PVP and ACFCeP

Table S5: Non-Linear isotherm rate constant and regression coefficient values for Langmuir, Freundlich, D-R and Temkin isotherms.

Isotherms Model	Parameter	Value for ACFCeP composite
Langmuir	q_{max} (mg/gm)	44.65
	K (L/mg)	1.20
	R^2	0.979
Freundlich	K_f (mg/g)	23.64
	n	0.212
	R^2	0.922
D-R	K_{DR}	0.388
	qm (mg/g)	38.17
	R^2	0.7069
Temkin	A (Unitless)	6.63
	K_T (L/mg)	40.27
	b (kg/mol)	373.69
	R^2	0.9678

Table S6 Non-Linear pseudo first order, pseudo second order and Elovich kinetic model parameters

Kinetic model and parameter	20 ppm	60 ppm	100 ppm
Experimental q_e (mg/g)	11.9081	32.5260	44.0642
Pseudo first order (PFO)			
q_e (mg/g)	11.6	31.6	41.9
K_1 (min^{-1})	0.234	0.212	0.0957
R^2	0.8989	0.9794	0.9356
Pseudo second order (PSO)			
q_e (mg/g)	11.9	32.9	46.6

K_2 (g mg^{-1}min^{-1})	0.06778	0.0173	0.00312
R^2	0.999	0.999	0.988
Elovich Kinetics model			
a (mg g^{-1}min^{-1})	42.95	507791.309	2.75E8
b (g mg^{-1})	0.141	0.52072	2.07
R^2	0.997	0.999	0.998

Table-S7: Linear pseudo first order, second order and Elovich equations for different concentration

Concentration (ppm)	Pseudo first order equation	Pseudo second order equation	Elovich Kinetic equation
20	y = -0.0253x+0.0766	y = 0.0838x+0.1031	y = 0.481x+9.7066
60	y = -0.0402x+1.8352	y = 0.0302x+0.0662	y = 1.9204x+23.9768
100	y = -0.0657x+3.9194	y = 0.0206x+0.2149	y = 7.0224x+12.9815
